# A national database analysis for factors associated with thyroid cancer occurrence

**DOI:** 10.1038/s41598-020-74546-3

**Published:** 2020-10-20

**Authors:** Joon-Hyop Lee, Sora Youn, Sohee Jung, Kwangsoo Kim, Young Jun Chai, Yoo Seung Chung, Won Seo Park, Kyu Eun Lee, Ka Hee Yi

**Affiliations:** 1 Department of Surgery, Gachon University College of Medicine, Gil Medical Center, Incheon, Korea; 2 Division of Clinical Bioinformatics, Biomedical Research Institute, Seoul National University Hospital, 71 Daehak-ro, Jongno-gu, Seoul, 03082 Korea; 3 Department of Surgery, Seoul National University Boramae Medical Center, 20 Boramaep-ro 5-gil, Dongjak-gu, Seoul, 07061 Korea; 4 Department of Surgery, Kyung Hee University School of Medicine, Seoul, Korea; 5 Department of Surgery, Seoul National University Hospital, Seoul, Korea; 6 Department of Internal Medicine, Seoul Metropolitan Government Seoul National University Boramae Medical Center, Seoul, Korea

**Keywords:** Thyroid cancer, Cancer epidemiology

## Abstract

In order to analyze the associations between thyroid cancer and environmental factors, we analyzed the national sample cohort representative of the entire population provided by the Korean National Health Insurance Service database record from 2006 to 2015. The cohort was categorized according to age, body mass index, income, residential areas, frequency of exercise, frequency of alcohol drinking, diet, presence or absence of hyperthyroidism, presence or absence of hypothyroidism, and smoking data. Age ≥ 55 years (HR 0.68, 95% CI 0.53–0.88), lower income (0.57, 0.40–0.80), and current smoking (0.69, 0.55–0.85) were associated with lower thyroid cancer occurrence among men. Body mass index (BMI) ≥ 25 kg/m2 (1.51, 1.26–1.82), higher income (1.44, 1.19–1.76), urban residence (1.24, 1.03–1.49), and presence of hypothyroidism (3.31, 2.38–4.61) or hyperthyroidism (2.46, 1.75–3.46) were associated with higher thyroid cancer occurrence among men. Age ≥ 55 years (0.63, 0.56–0.71), moderate alcohol drinking (0.87, 0.77–0.99), and current smoking (0.56, 0.37–0.85) were associated with lower thyroid cancer occurrence among women. BMI ≥ 25 kg/m2 (1.41, 1.26–1.57), frequent exercise (1.21, 1.07–1.36), higher income (1.18, 1.06–1.32), urban residence (1.17, 1.06–1.29), and presence of hypothyroidism (1.60, 1.40–1.82) or hyperthyroidism (1.38, 1.19–1.61) were associated with higher thyroid cancer occurrence among women. In conclusion, age ≥ 55 years and current smoking were associated with lower thyroid cancer occurrence, while BMI ≥ 25 kg/m2, higher income, urban residence, hypothyroidism, and hyperthyroidism were associated with higher occurrence in both men and women.

## Introduction

Thyroid cancer is globally the most common endocrine malignancy, and its incidence has increased worldwide^1–4^. However, the causes of thyroid cancer, apart from exposure to radiation during childhood, have not been elucidated^1^. This is in contrast with the well-documented causal effects of lifestyle or environmental factors such as smoking, alcohol drinking, and high-sodium diet on cancers of the lung, liver, and stomach, respectively. Many environmental factors have been proposed to be associated with thyroid cancer occurrence; however, this remains controversial^1^.

For example, iodine uptake has been linked with the development of thyroid cancer; however, the evidence is inconclusive^5,6^. Likewise, reported associations between thyroid cancer and environmental factors, such as obesity, physical activity, socio-economic status (SES), alcohol drinking, and smoking, differ among studies with small and biased population selection^7–10^. Moreover, these studies investigated the association of each factor with thyroid cancer separately and thus neglected the confounding influence among these factors.

In recent years, large-scale databases of national insurance systems have become publicly available in several countries, including South Korea^11,12^. Analyzing such databases allows investigators not only to comprehensively assess the associations of multiple environmental factors with a disease and their confounding influence, but also to perform such analyses using a population that is representative of an entire nation. Therefore, we comprehensively analyzed the associations between thyroid cancer and environmental factors by extracting a thyroid cancer patient group and a control non-cancer group from the nation-wide insurance database.

## Results

Among the 1,021,208 individuals in the Korean National Health Insurance Service (KNIHS) national sample cohort database, 176,387 and 104,588 received health examinations in 2006 and 2007, respectively. During the selection process, 2106 eligible patients with thyroid cancer and 232,680 participants without a history of thyroid cancer were enrolled as cases and controls, respectively. The median follow-up period for the case and control groups were 4.79 and 9.07 years respectively. The baseline characteristics of the study population are shown in Table 2. The χ^2^-test indicated that all variables significantly differed between cases and controls (*p* < 0.05, Table 1).

**Table 1:** Baseline characteristics of the study population.

Variable	Total	Control cohort No. (%)	Thyroid cancer cohort No. (%)	*p* value
Total	234,786	232,680 (99.0)	2106 (1.0)	
**Sex**				< 0.001
Men	124,318	123,857 (99.6)	461 (0.4)	
Women	110,468	108,823 (98.5)	1645 (1.5)	
**Age (years)**				< 0.001
< 55	170,753	169,129 (99.0)	1624 (1.0)	
≥ 55	64,033	63,551 (99.2)	482 (0.8)	
**BMI (kg/m**^**2**^**)**				0.001
< 25	162,970	161,576 (99.1)	1394 (0.9)	
≥ 25	71,816	71,104 (99.0)	712 (1.0)	
**Exercise**				0.01
< 3 times per week	191,097	189,429 (99.1)	1668 (0.9)	
≥ 3 times per week	43,689	43,251 (99.0)	438 (1.0)	
**Income**				< 0.001
Middle class	96,125	95,342 (99.2)	783 (0.8)	
Lower class	49,807	49,422 (99.2)	385 (0.8)	
Upper class	88,854	87,916 (98.9)	938 (1.1)	
**Residential area**				< 0.001
Others	127,234	126,194 (99.2)	1040 (0.8)	
City	107,552	106,486 (99.0)	1066 (1.0)	
**Hypothyroidism**				< 0.001
No	220,169	218,392 (99.2)	1777 (0.8)	
Yes	14,617	14,288 (97.7)	329 (2.3)	
**Hyperthyroidism**				< 0.001
No	222,220	220,360 (99.2)	1860 (0.8)	
Yes	12,566	12,320 (98.0)	246 (2.0)	
**Alcohol drinking**				< 0.001
Never	124,607	123,198 (98.9)	1409 (1.1)	
< 3 times per week	89,835	89,209 (99.3)	626 (0.7)	
≥ 3 times per week	20,344	20,273 (99.7)	71 (0.3)	
**Diet**				< 0.001
Vegetarian	45,116	44,615 (98.9)	501 (1.1)	
Well-balanced	176,606	175,105 (99.2)	1501 (0.8)	
Meat	13,064	12,960 (99.2)	104 (0.8)	
**Smoking**				< 0.001
Never smoker	161,157	159,323 (98.9)	1834 (1.1)	
Former smoker	16,447	16,351 (99.4)	96 (0.6)	
Current smoker	57,182	57,006 (99.7)	176 (0.3)	

The results of multivariable survival analysis for men and women are shown in Supplement 1 and 2. Age ≥ 55 years (HR 0.68, 95% CI 0.53–0.88), lower income (HR 0.57, 95% CI 0.40–0.80), and current smoking (HR 0.69, 95% CI 0.55–0.85) were associated with lower thyroid cancer occurrence among men. BMI ≥ 25 kg/m^2^ (HR 1.51, 95% CI 1.26–1.82), higher income (HR 1.44, 95% CI 1.19–1.76), urban residence (HR 1.24, 95% CI 1.03–1.49), and presence of hypothyroidism (HR 3.31, 95% CI 2.38–4.61) or hyperthyroidism (HR 2.46, 95% CI 1.75–3.46) were associated with higher thyroid cancer occurrence among men (*p* < 0.05, Supplement 1).

Age ≥ 55 years (HR 0.63, 95% CI: 0.56–0.71), moderate alcohol drinking (HR 0.87, 95% CI 0.77–0.99), and current smoking (HR 0.56, 95% CI 0.37–0.85) were associated with lower thyroid cancer occurrence among women. BMI ≥ 25 kg/m^2^ (HR 1.41, 95% CI 1.26–1.57), frequent exercise (HR 1.21, 95% CI 1.07–1.36), higher income (HR 1.18, 95% CI 1.06–1.32), urban residence (HR 1.17, 95% CI 1.06–1.29), and presence of hypothyroidism (HR 1.60, 95% CI 1.40–1.82) or hyperthyroidism (HR 1.38, 95% CI 1.19–1.61) were associated with higher thyroid cancer occurrence among women (*p* < 0.05, Supplement 2).

Development of thyroid cancer according to smoking status was analyzed separately for each gender because of the demographical difference (difference in incidence of thyroid cancer, female prone) that may allude to heterogeneity of thyroid cancer between the two genders, and because the variables may be exhibited differently between the two genders. For example, social taboos against women smoking tobacco may elicit different response patterns in health survey between the genders (Table 2). The percentage of current smokers was 43.6% among men and 2.7% among women. During the 2074182.2 person-years of follow-up, 2106 participants developed thyroid cancer and received surgery for it (incidence rate, 1.01 per 1000 person-years). For men, only current smoking was significantly associated with a decreased thyroid cancer risk when adjusted for multiple variables (HR 0.69, 95% CI 0.55–0.85). Former smoking was not associated with an increased risk of developing thyroid cancer compared with never smoking. Likewise, current smoking was associated with a decreased risk of thyroid cancer among women in the multivariable adjusted model, whereas former smoking was not.

**Table 2:** Development of thyroid cancer according to smoking status.

Sex	Smoking status	Person-years of follow-up	Incident cases	Incidence density (per 1000 person-years)	Multivariable adjusted HR (95% CI)
Men	Never smoker	484,157.9	228	0.47	1.00 (reference)
	Former smoker	136,746.4	80	0.59	1.13 (0.87–1.46)
	Current smoker	480,880.1	153	0.32	0.69 (0.55–0.85)
Women	Never smoker	937,322.1	1606	1.71	1.00 (reference)
	Former smoker	9,343.2	16	1.71	1.05 (0.64–1.73)
	Current smoker	25,732.5	23	0.89	0.56 (0.37–0.85)

The multivariable adjusted model demonstrated an association between pack-years of smoking and development of thyroid cancer among men (Supplement 3). The risk of developing thyroid cancer was significantly lower in each of the pack-years groups than in never smokers. However, pack-years of smoking was not associated with thyroid cancer occurrence among women.

## Discussion

According to our study, younger age, BMI ≥ 25 kg/m^2^, higher income, urban residence, and presence of hypothyroidism or hyperthyroidism, were associated with higher thyroid cancer occurrence in both genders, while less physical activity, moderate alcohol drinking and current smoking were associated with lower thyroid cancer occurrence in only women. Pack-years of smoking was associated with a decreased risk of thyroid cancer in men, but not in women.

This study is the first to comprehensively analyze the effects of lifestyle and environmental factors on thyroid cancer occurrence using a national database. Previous national database studies investigated the impact of individual environmental factors on thyroid cancer occurrence. Using the National Health Insurance database of Taiwan, two separate groups concluded that the presence of hyperthyroidism, higher SES, and urban residence are associated with an increased risk of thyroid cancer occurrence^12,13^. Likewise, a 7-year follow-up study of the KNHIS database demonstrated that higher BMI is associated with an increased risk of thyroid cancer^11,14^. However, these studies investigated the effects of individual factors, whereas the present study conducted multivariable adjusted analysis of epidemiologic factors to reduce confounding effects. Furthermore, insurance databases are designed for cost-claim analysis, not for research purposes, which may lead to questions about the validity of thyroid cancer diagnosis^15^. However, because of the reimbursement policies, diagnostic codes for cancer tend to be more accurate than those for benign conditions^16^. In addition, we strengthened the diagnostic accuracy by requiring registration of multiple cancer codes (to exclude unconfirmed diagnosis) followed by a thyroid operation code. Therefore, diagnosis of thyroid cancer in our study can be considered reliable.

National database analysis has great significance because it is representative of an entire population or nation; therefore, the generalizability of the data is more acceptable in comparison with individual studies. Our results are representative of the South Korean population and are in accordance with previous studies^14–18^. Young age, high SES, urban residence, obesity, and hypothyroidism have been linked to an increased risk of thyroid cancer occurrence in both national database and individual studies. Although young age is well-documented to be associated with lower thyroid cancer mortality^19^, it is not always associated with lower thyroid cancer occurrence. Multiple genetic alterations are found more frequently in younger patients^17^. For example, *RET/PTC1* rearrangement, which is a key somatic genetic alteration in papillary thyroid cancer development, occurs more frequently in younger patients^18^. Such factors may underlie national database-based results demonstrating an increased risk of papillary thyroid cancer lymph node metastasis in younger patients^20^.

The associations of thyroid cancer with higher SES and urban residence can be attributed to easy access to medical healthcare^21^. Individuals who reside in cities earn more and tend to receive frequent medical check-ups, which may explain the increase in thyroid cancer occurrence^22,23^. Likewise, obesity has been associated with a higher occurrence of thyroid cancer in many studies^11^. Although the underlying mechanism is not fully understood, insulin resistance or diabetes, which is associated with obesity, may be a risk factor for tumor development^24^. Hyperthyroidism and hypothyroidism have also been associated with thyroid cancer. Thyroid-stimulating antibody may affect proto-oncogenes such as *RET* and *TRK* during the development of thyroid cancer in hyperthyroidism, and an increased thyroid-stimulating hormone level may lead to nodule and cancer growth in patients with hypothyroidism^25^. Furthermore, frequent ultrasonography performed in these patients may increase the chance of detecting thyroid cancer^26^.

The effect of smoking on thyroid cancer has been extensively investigated in many individual studies. In general, active cigarette smoking is one of the most well-known causes of cancer. While some data support the carcinogenic effect of smoking in thyroid cancer^27^, others demonstrate no significant effect ^28,29^ and some even indicate that cigarette smoking protects against thyroid cancer^30,31^. Subsequently, a meta-analysis demonstrated that current smokers are less likely to be diagnosed with thyroid cancer than non-smokers^7^ and the general opinion is in accordance with this result. The theory that smoking exerts a protective effect for thyroid cancer is based on the finding that cigarette extracts exhibit properties similar to that of thyroid hormones and thus may act as thyroid hormone receptor partial agonists^32^. This may mimic a ‘thyroid stimulating hormone suppression effect’ which would lead to decreased thyroid gland stimulation and in turn less of tumorigenesis. Our study is the first to confirm this association using a national database, which strengthens the generalizability of the association between current smoking and decreased thyroid cancer occurrence.

Although national insurance databases have their strengths, they can only reveal associations, not causality, due to their inherent quality. Such causal relationships must be demonstrated in individual studies investigating the mechanisms underlying thyroid carcinogenesis. Furthermore, clinical information, such as cancer stage, type of thyroid cancer (i.e. medullary or anaplastic cancer), and laboratory data (e.g., thyroid function test results), were not included in this study. In addition, the questionnaires provided by the participants did not include any information on the time duration of the variables. This was complicated by the fact that questionnaires were reported in two different formats (never/former/current vs. pack-year format) obtained through patients’ own reports. Consequently, subgroup analyses pertaining to such clinical and temporal information could not be conducted. Finally, the non-linear effect of smoking pack-years and the different effect on both genders could not be explained by our data. Perhaps additional data must be further analyzed through a meticulously designed study in order to elucidate the effect of smoking pack-years on thyroid cancer.

In conclusion, our study provides representative data about the environmental factors that are associated with thyroid cancer occurrence. Age ≥ 55 years and current smoking were associated with lower thyroid cancer occurrence, while ≥ BMI 25 kg/m^2^, higher income, urban residence, hypothyroidism, and hyperthyroidism were associated with higher thyroid cancer occurrence in both men and women. Further individual studies focusing on the mechanism underlying thyroid carcinogenesis are needed to elucidate the causality between these factors and thyroid cancer occurrence.

## Methods

### Database

Around 97% of the South Korean population is enrolled in national health insurance, and the KNHIS has publicly disclosed its national medical care claims information database. This database contains reimbursement claims from all medical facilities in the nation, which are paired with personal information data including SES, medical procedures, diagnostic codes, prescription drugs, information about the medical facility, inpatient and outpatient care medical costs, and dental services. There were 48,222,537 individuals who had Korean citizenship and maintained health insurance membership in 2006. Among them, the KNHIS selected 1,021,208 (approximately 2%) individuals in accordance with proportionate stratified random sampling based on 2142 strata (consisting of age, gender, residential area, and income level) to form the nationally representative sample database (national sample cohort). All of their medical claim information was prospectively collected up to 2015, and newborn cohorts were annually added to replace the deceased and to maintain the representability of the national sample cohort. Personal and sensitive information was replaced with information identifiers. We acquired institutional review board approval prior to conducting this analysis (No. GCIRB2019-318).

### Study population

**Figure 1:**
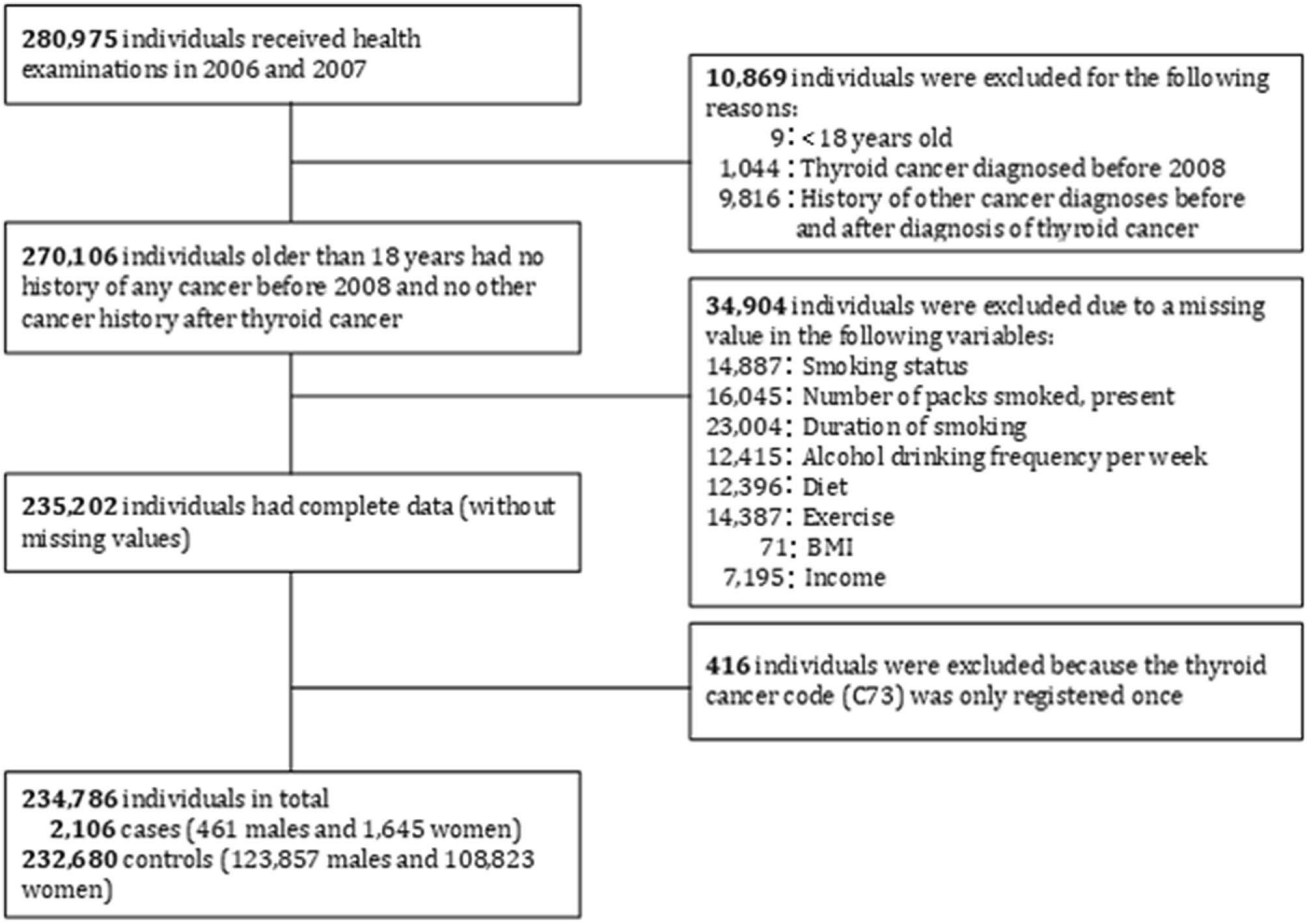
Data flow diagram of the cohort study.

This was a retrospective study of a prospective national cohort: the KNHIS national sample cohort. Individuals who made any healthcare examination claims in 2006 or 2007 were enrolled for analysis. Only medical claim information from 2006 was evaluated for individuals who made claims in both years. Individuals who had the thyroid cancer code (Korean Classification of Disease (KCD) code C73) registered more than once and a thyroid surgery code (operation codes that included all types of thyroidectomies: P4551, P4552, P4554, or P4561) registered subsequently were categorized as the thyroid cancer group. Individuals who had never had the thyroid cancer code registered were classified as the control group. Individuals who satisfied at least one of the following criteria were excluded from the analysis: (1) minors younger than 18 years old at the time of enrollment: (2) history of thyroid cancer diagnosis before enrollment or of any other cancer diagnosis regardless of the time period; and (3) variables containing missing values (Fig. 1).

### Data collection

The study population was categorized according to age, body mass index (BMI), income, residential areas, frequency of exercise, frequency of alcohol drinking, diet, presence or absence of hyperthyroidism, presence or absence of hypothyroidism, and smoking data (smoking status and pack-years) at the time of examination (i.e. 2006 ~ 2007). The categorization was performed as follows: age (< 55 years and ≥ 55 years), BMI (< 25 kg/m^2^: normal, and ≥ 25 kg/m^2^: overweight), income (middle class: 4th–7th decile, upper class: 1st–3rd decile, and lower class: 8th–10th decile), residential area (metropolitan cities and others), frequency of exercise (< 3 times per week and ≥ 3 times per week), alcohol drinking (never, < 3 times per week and ≥ 3 times per week), diet (vegetarian, meat only, and both), and smoking status (never, former, and current smokers). Smoking pack-years was calculated as the number of packs smoked per day multiplied by the number of years of previous or current smoking among current smokers. The duration of smoking was provided as categorical variables; therefore, the median of each category was used to calculate the pack-years, which were subsequently categorized into 0, < 10, 10–19.9, and ≥ 20 for men and 0, < 5, 5–9.9, and ≥ 10 for women (Table 3). The study endpoints were defined as all-cause mortality or the first incidence of thyroid cancer up to 2015.

**Table 3:** Variables in the cohort study.

Variable	Categories
Age (years)	1: < 55 years old
	2: ≥ 55 years old
BMI (kg/m^2^)	1: < 25 (underweight and normal)
	2: ≥ 25 (overweight)
Exercise	1: 0–2 times per week
	2: > 3 times per week
Income	1: Middle class (4th–7th quantile)
	2: Lower class
	3: Upper class
Residential area	1: Metropolitan areas (Seoul, Gyeonggi-do, and megalopolis)
	2: Others
Hypothyroidism	0: No
	1: Yes
Hyperthyroidism	0: No
	1: Yes
Alcohol drinking	1: Never
	2: < 3 times per week
	3: ≥ 3 times per week
Diet	1: Vegetarian
	2: Well-balanced
	3: Meat
Smoking	1: Never smoker
	2: Former smoker
	3: Current smoker
Pack-years	(Packs smoked per day)$\times$(years as a smoker)
Period (days)	Number of days until diagnosis of thyroid cancer or death
Diagnosis of thyroid cancer	0: no C73 or surgery code
	1: Registration of the C73 code more than twice and surgery codes (P4551, P4553, P4554, and P4561)
Number of packs smoked, present	1: 0.5 pack
	2: 0.5–1 pack
	3: 1–2 packs
	4: > 2 packs
Duration of smoking, past and present	1: < 5 years
	2: 5–9 years
	3: 10–19 years
	4: 20–29 years
	5: > 30 years

### Statistical analysis

Independent variables were compared between the thyroid cancer and control groups using the χ2-test and further multivariate analysis was conducted with Cox regression. The incidence of thyroid cancer significantly differs according to gender; therefore, analyses were performed separately for men and women. All variables were compared according to the smoking status using the χ^2^-test for binomial variables. Multivariate analysis was conducted using the Cox regression model.

For smoking, the incidence density (per 1000 person-years) was obtained by calculating the ratio of the number of incident cases by person-years to the sum of the observation periods of all persons in each group. Multivariate Cox proportional hazards regression models were used to calculate the incidence, survival rate, hazard ratio (HR), and 95% confidence interval (CI) of smoking status and pack-years. The model was adjusted for the following variables: age, BMI, exercise, income, residential area, hypothyroidism, hyperthyroidism, alcohol drinking, and diet (Table 1). All statistical analyses were performed using R Studio, version 1.0.136, with a two-sided significance level of 0.05.

## Supplementary information

Supplementary Information.

## References

[1] Pellegriti G, Frasca F, Regalbuto C, Squatrito S, Vigneri R (2013). Worldwide increasing incidence of thyroid cancer: update on epidemiology and risk factors. J. Cancer Epidemiol..

[2] Olson E, Wintheiser G, Wolfe KM, Droessler J, Silberstein PT (2019). Epidemiology of thyroid cancer: a review of the national cancer database, 2000–2013. Cureus.

[3] Atamari-Anahui N (2019). National trends in prevalence and mortality rates of thyroid cancer using data from the Ministry of Health of Peru. Medwave.

[4] Du L (2019). Incidence and mortality of thyroid cancer in China, 2008–2012. Chin. J. Cancer Res..

[5] Lee JH (2017). Relationship between iodine levels and papillary thyroid carcinoma: a systematic review and meta-analysis. Head Neck.

[6] Lee JH (2018). Case-control study of papillary thyroid carcinoma on urinary and dietary iodine status in South Korea. World J. Surg..

[7] Cho YA, Kim J (2014). Thyroid cancer risk and smoking status: a meta-analysis. Cancer Causes Control (CCC).

[8] Hong SH, Myung SK, Kim HS, Korean Meta-Analysis Study, G (2017). Alcohol intake and risk of thyroid cancer: a meta-analysis of observational studies. Cancer Res. Treat..

[9] Fiore M (2019). Physical activity and thyroid cancer risk: a case-control study in Catania (South Italy). Int. J. Environ. Res. Public Health.

[10] Guay B, Johnson-Obaseki S, McDonald JT, Connell C, Corsten M (2014). Incidence of differentiated thyroid cancer by socioeconomic status and urban residence: Canada 1991–2006. Thyroid.

[11] Son H, Lee H, Kang K, Lee I (2018). The risk of thyroid cancer and obesity: a nationwide population-based study using the Korea National Health Insurance Corporation cohort database. Surg. Oncol..

[12] Yeh NC (2013). Hyperthyroidism and thyroid cancer risk: a population-based cohort study. Exp. Clin. Endocrinol. Diabetes.

[13] Liu FC (2017). Nationwide cohort study on the epidemiology and survival outcomes of thyroid cancer. Oncotarget.

[14] Kim M, Yim S, Lee J, Jo Y (2018). Association between obesity and tumor size in patients with papillary thyroid cancer. J. Endocr. Surg..

[15] Cho GJ (2019). Risk of adverse obstetric outcomes and the abnormal growth of offspring in women with a history of thyroid cancer. Thyroid.

[16] Park, B. J., Sung, J. H., Park, K. D., Seo, S. W. & Kim, S. W. *Strategies to Improve the Validity of Diagnostic Codes of National Health Insurance Claims Data* 118–119 (Health Insurance Review and Assessment Services, Seoul, 2002).

[17] Moses W (2010). Multiple genetic alterations in papillary thyroid cancer are associated with younger age at presentation. J. Surg. Res..

[18] Nikiforov YE (2002). RET/PTC rearrangement in thyroid tumors. Endocr Pathol.

[19] Haugen BR (2016). 2015 American Thyroid Association Management Guidelines for adult patients with thyroid nodules and differentiated thyroid cancer: The American Thyroid Association Guidelines Task Force on thyroid nodules and differentiated thyroid cancer. Thyroid.

[20] Wang J (2018). Young age increases the risk of lymph node positivity in papillary thyroid cancer patients: a SEER data-based study. Cancer Manag. Res..

[21] Yang S, Chang H, Lim C (2019). Analysis of correlation between thyroid cancer incidence and socioeconomic status using 10-year sample cohort database. J. Endocr. Surg..

[22] Ahn HS, Kim HJ, Welch HG (2014). Korea's thyroid-cancer "epidemic"–screening and overdiagnosis. N. Engl. J. Med..

[23] Vaccarella S (2015). The impact of diagnostic changes on the rise in thyroid cancer incidence: a population-based study in selected high-resource countries. Thyroid.

[24] Rezzonico JN, Rezzonico M, Pusiol E, Pitoia F, Niepomniszcze H (2009). Increased prevalence of insulin resistance in patients with differentiated thyroid carcinoma. Metab. Syndr. Relat. Disord..

[25] Liang L (2019). Association of benign thyroid diseases with thyroid cancer risk: a meta-analysis of prospective observational studies. J. Endocrinol. Invest..

[26] Walsh JP (2016). Managing thyroid disease in general practice. Med. J. Aust..

[27] Sokic SI (1994). Risk factors for thyroid cancer. Neoplasma.

[28] Iribarren C, Haselkorn T, Tekawa IS, Friedman GD (2001). Cohort study of thyroid cancer in a San Francisco Bay area population. Int. J. Cancer.

[29] Guignard R, Truong T, Rougier Y, Baron-Dubourdieu D, Guenel P (2007). Alcohol drinking, tobacco smoking, and anthropometric characteristics as risk factors for thyroid cancer: a countrywide case-control study in New Caledonia. Am. J. Epidemiol..

[30] Mack WJ (2003). A pooled analysis of case-control studies of thyroid cancer: cigarette smoking and consumption of alcohol, coffee, and tea. Cancer Causes Control.

[31] Kitahara CM (2012). Cigarette smoking, alcohol intake, and thyroid cancer risk: a pooled analysis of five prospective studies in the United States. Cancer Causes Control.

[32] Boelaert K (2006). Serum thyrotropin concentration as a novel predictor of malignancy in thyroid nodules investigated by fine-needle aspiration. J. Clin. Endocrinol. Metab..

