# Supplementary material for: A national database analysis for factors associated with thyroid cancer occurrence
Source: Sci Rep. 2020 Oct 20;10:17791. doi: 10.1038/s41598-020-74546-3 (PMC7576121; doi:10.1038/s41598-020-74546-3)
Supplement: Supplementary file 1 — Supplementary Information. [file 41598_2020_74546_MOESM1_ESM.docx]

*Title page*

**A national database analysis for factors associated with thyroid cancer occurrence**

Joon-Hyop Lee, MD^1+^, Sora Youn, MS^2+^, Sohee Jung, BA^2^, Kwangsoo Kim, PhD^2*^, Young Jun Chai, MD, PhD^3*^, Yoo Seung Chung, MD, PhD^1^, Won Seo Park, MD, PhD^4^, Kyu Eun Lee, MD, PhD^5^, Ka Hee Yi, MD, PhD^6^

^1^Department of Surgery, Gachon University College of Medicine, Gil Medical Center, Incheon, Korea

^2^Division of Clinical Bioinformatics, Biomedical Research Institute, Seoul National University Hospital, Seoul, Korea

^3^Department of Surgery, Seoul Metropolitan Government Seoul National University Boramae Medical Center, Seoul, Korea

^4^Department of Surgery, Kyung Hee University School of Medicine, Seoul, Korea

^5^Department of Surgery, Seoul National University Hospital, Seoul, Korea

^6^Department of Internal Medicine, Seoul Metropolitan Government Seoul National University Boramae Medical Center, Seoul, Korea

Supplement 1. Multivariate cox regression for men

| **Variable** | | **HR (95% CI)** | **p-value** |  |
| --- | --- | --- | --- | --- |
| **Age (years)** |  |  |  |  |
| <55 | 1.00 (reference) |  |  |  |
| ≥55 | 0.68 (0.53–0.88) | 0.003 |  |  |
| **BMI (kg/m^2^)** |  |  |  |  |
| <25 | 1.00 (reference) |  |  |  |
| ≥25 | 1.51 (1.26–1.82) | <0.001 |  |  |
| **Exercise** |  |  |  |  |
| <3 times per week | 1.00 (reference) |  |  |  |
| ≥3 times per week | 0.96 (0.76–1.21) | 0.732 |  |  |
| **Income** |  |  |  |  |
| Middle class | 1.00 (reference) |  |  |  |
| Lower class | 0.57 (0.40–0.80) | 0.001 |  |  |
| Upper class | 1.44 (1.19–1.76) | <0.001 |  |  |
| **Residential area** |  |  |  |  |
| Others | 1.00 (reference) |  |  |  |
| City | 1.24 (1.03–1.49) | 0.021 |  |  |
| **Hypothyroidism** |  |  |  |  |
| No | 1.00 (reference) |  |  |  |
| Yes | 3.31 (2.38–4.61) | <0.001 |  |  |
| **Hyperthyroidism** |  |  |  |  |
| No | 1.00 (reference) |  |  |  |
| Yes | 2.46 (1.75–3.46) | <0.001 |  |  |
| **Alcohol drinking** |  |  |  |  |
| Never | 1.00 (reference) |  |  |  |
| <3 times per week | 1.12 (0.91–1.39) | 0.285 |  |  |
| ≥3 times per week | 0.77 (0.55–1.08) | 0.135 |  |  |
| **Diet** |  |  |  |  |
| Vegetarian | 1.00 (reference) |  |  |  |
| Well-balanced | 0.92 (0.71–1.20) | 0.549 |  |  |
| Meat | 1.37 (0.93–2.02) | 0.109 |  |  |
| **Smoking** |  |  |  |  |
| Never smoker | 1.00 (reference) |  |  |  |
| Former smoker | 1.13 (0.87–1.46) | 0.374 |  |  |
| Current smoker | 0.69 (0.55–0.85) | <0.001 |  |  |

Supplement 2. Multivariate cox regression for women

| **Variable** | | **HR (95% CI)** | **p-value** | |
| --- | --- | --- | --- | --- |
| **Age (years)** |  |  |  |  |
| <55 | 1.00 (reference) |  |  |  |
| ≥55 | 0.63 (0.56–0.71) | <0.001 |  |  |
| **BMI (kg/m^2^)** |  |  |  |  |
| <25 | 1.00 (reference) |  |  |  |
| ≥25 | 1.41 (1.26–1.57) | <0.001 |  |  |
| **Exercise** |  |  |  |  |
| <3 times per week | 1.00 (reference) |  |  |  |
| ≥3 times per week | 1.21 (1.07–1.36) | <0.002 |  |  |
| **Income** |  |  |  |  |
| Middle class | 1.00 (reference) |  |  |  |
| Lower class | 0.92 (0.80–1.05) | 0.202 |  |  |
| Upper class | 1.18 (1.06–1.32) | 0.001 |  |  |
| **Residential area** |  |  |  |  |
| Others | 1.00 (reference) |  |  |  |
| City | 1.17 (1.06–1.29) | 0.001 |  |  |
| **Hypothyroidism** |  |  |  |  |
| No | 1.00 (reference) |  |  |  |
| Yes | 1.60 (1.40–1.82) | <0.001 |  |  |
| **Hyperthyroidism** |  |  |  |  |
| No | 1.00 (reference) |  |  |  |
| Yes | 1.38 (1.19–1.61) | <0.001 |  |  |
| **Alcohol drinking** |  |  |  |  |
| Never | 1.00 (reference) |  |  |  |
| <3 times per week | 0.87 (0.77–0.99) | 0.028 |  |  |
| ≥3 times per week | 0.87 (0.58–1.30) | 0.494 |  |  |
| **Diet** |  |  |  |  |
| Vegetarian | 1.00 (reference) |  |  |  |
| Well-balanced | 0.90 (0.81–1.00) | 0.065 |  |  |
| Meat | 0.89 (0.66–1.16) | 0.359 |  |  |
| **Smoking** |  |  |  |  |
| Never smoker | 1.00 (reference) |  |  |  |
| Former smoker | 1.05 (0.64–1.73) | 0.833 |  |  |
| Current smoker | 0.56 (0.37–0.85) | 0.007 |  |  |

Supplement 3. Development of thyroid cancer according to pack-years

| **Sex** | **Smoking status**  **(pack-years)** | **Person-years** | **Incident cases** | **Incidence density (per 1,000 person-years)** | **Multivariable adjusted HR**  **(95% CI)** |
| --- | --- | --- | --- | --- | --- |
| **Men** | **0** | 620,904.2 | 308 | 0.50 | 1.00 (reference) |
|  | **<10** | 136,963.6 | 39 | 0.28 | 0.57 (0.41–0.80) |
|  | **10–19.9** | 203,014.6 | 71 | 0.35 | 0.71 (0.55–0.93) |
|  | **≥20** | 140,901.9 | 43 | 0.31 | 0.67 (0.48–0.92) |
| **Women** | **0** | 946,665.4 | 1,622 | 0.71 | 1.00 (reference) |
|  | **<5** | 13,346.9 | 14 | 1.05 | 0.64 (0.38–1.09) |
|  | **5–9.9** | 4,063.6 | 2 | 0.49 | 0.31 (0.08–1.24) |
|  | **≥10** | 8,322.0 | 7 | 0.84 | 0.55 (0.26–1.17) |
